# Impact of the COVID-19 pandemic on visits to the hospital emergency service in two hospitals in Spain, from March 14, 2020 to June 21, 2020

**DOI:** 10.1007/s11739-023-03328-2

**Published:** 2023-06-12

**Authors:** Iria Sanlés González, Héctor Alonso Valle, Laura Grimal Abejez, Anna Carreres Molas, Jéssica Alonso-Molero, Trinidad Dierssen-Sotos, Inés Gómez-Acebo

**Affiliations:** 1 Department of Emergency Medicine, Germans Trias i Pujol University Hospital, c/ Canyet, 08916 Badalona, Spain; 2 Department of Emergency Medicine, Marqués de Valdecilla University Hospital, Santander, Spain; 3 Medicine School, Cantabria University, Santander, Spain; 4 IDIVAL Valdecilla Health Research Institute, Santander, Spain; 5 CIBER Epidemiology and Public Health (CIBERESP), Madrid, Spain

**Keywords:** COVID-19, Hospital emergency departments, Pandemics, ICD-10

## Abstract

This study will describe trends in the use of emergency departments before and after the Spanish State of Alarm, especially in pathologies not directly related to this infection. A cross-sectional study was conducted of all visits to the emergency departments in two third-level hospitals in two Spanish communities during the Spanish State of Alarm, compared with the same period of the previous year. The variables collected included the day of the week, the time of the visit, the duration of the visit, the final destination of the patients (home, admission to a conventional hospitalization ward, admission to the intensive care unit, and death), and the diagnosis at discharge according to the International Classification of Diseases 10th Revision. During the Spanish State of Alarm period, an overall decrease in care demand of 48% was observed, which reached 69.5% in pediatric emergency departments. We also saw a drop of between 20 and 30% in time-dependent pathologies (heart attack, stroke, sepsis, poisoning). The decrease in overall attendance in the emergency departments and absence of serious pathologies, such as time-dependent diseases, observed during the Spanish State of Alarm compared to the previous year highlights the need to strengthen the messages addressed to the population to encourage them to seek care without delay in case of alarming symptoms and reduce the high morbidity and mortality rate if the diagnosis is delayed.

## Introduction

From March 14 to June 21, 2020, coinciding with the first wave of COVID-19, Spain declared the Spanish State of Alarm (SSA) to combat this disease. Its restrictions and limitations affected mobility and non-essential travel, the closure of training and business entities, outdoor activities, social distancing, hand hygiene measures, and the use of gloves and masks [1, 2].

The state impact was serious, Spain was the third country with the highest number of COVID-19 cases diagnosed in Europe. However, there are differences between the different autonomous communities, both in the number of people affected and in the mortality rate. The Ministry of Health reported at the end of this period a total of 246,272 cases and 28,323 deaths. The most affected communities were Madrid, Catalonia, and Castilla y León [3]. In addition to the social impact, the health impact stands out above all, in particular the impact on the emergency departments (EDs). Due to the care overload secondary a greater severity and complexity of the pathologies treated, an effect already observed in the context of other epidemics such as the severe acute respiratory syndrome (SARS) or Middle East respiratory syndrome (MERS) [4, 5]. During this first wave, while the number of people hospitalized with COVID-19 increased, the number of visits to the ED decreased, especially those with unrelated COVID-19 pathologies, which could have led to an increase of global morbidity and mortality. Between these pathologies, we highlight those that are most life threatening, especially the diseases categorized within the emergency codes [6–10].

To quantify the effect of COVID-19 on the global care dynamics of two EDs with different disease incidences on the drop in the total number of care visits and serious pathologies during the SSA, we compared the volume of visits in this period with the previous year.

## Methods

This is a retrospective and cross-sectional study of two third-level hospitals in two Spanish communities. The HUMV, located in Cantabria, has 907 beds and covers a population of 300,000 inhabitants. The number of patients diagnosed with COVID-19 during the SSA in this community was 2344 cases. The HUGTIP located in Catalonia, and one of the reference hospitals for patients with severe COVID-19 during the EAE, has 643 beds and covers a population of more than 800,000 inhabitants. The number of patients diagnosed with COVID-19 during the SSA in this community was 60,645.

All patients who attended the EDs of both hospitals between March 14 and June 21, 2020 (pandemic or SSA period) were selected and compared with the same period of the previous year (comparison period). The population analyzed included both children and adults to have a broader view of EDs attendance. They were differentiated by pediatric patients, < 18 years old; gynecological and obstetric patients; general emergency patients, > 18 years old. Visits to EDs without recorded data and without established diagnoses were excluded.

The data to assess trends in Ed visits during the SSA were collected from the minimum basic data set of both centers. The collected data elements included the following: sociodemographic characteristics, including age and sex, the characteristics of the visits to the ED such as the day of the week, the time of visit by work shifts (08:00–15:00, 15:00–22:00, 22:00–08:00), the average stay, the final destination (home, hospital admission, intensive care unit (ICU) admission, and death), and the discharge diagnosis according to the 10th International Classification of Diseases (ICD-10) [11]. Patients considered to have died in the ED were both those registered as dead and those who died the first 24 h after hospital admission. In the subgroup analysis, we considered six age groups: “pediatric” from 0 to 17 years; “youth” from 18 to 44 years; “adults” from 45 to 59 years; “older adults” from 60 to 74 years; “elderly” from 75 to 89 years; and “long-lived elderly” 90 years or older.

The different phases of the SSA were analyzed to see the impact of the different rules of restriction and/or de-escalation on ED assistance. Four periods classified were: (a) phase 0 or home confinement situation: from March 14 to May 10; (b) phase 1 or initial de-escalation phase: from May 11 to 24; (c) phase 2 or middle de-escalation phase: from May 25 to June 7; (d) phase 3 or final de-escalation phase: from June 8 to 21.

To analyze the time-dependent processes, codes were defined according to the ICD-10: (1) serious acute cardiovascular diseases: myocardial infarction (MI) I21–I24 and stroke I60–I63; (2) sepsis: A41 and R65.21; (3) acute intoxications: F10–F19 and T36–T50.

### Analysis

The daily number of ED visits and their characteristics were examined during the SSA and the same period of the previous year. The mean ± standard deviation was used for continuous variables and percentages for categorical variables. The proportions’ comparison was made with the Chi-square test. Mean comparisons were made with Student’s *t* test.

The change in the average number of visits during the SSA and the comparison period was calculated as the average difference in total visits between the two periods (SSA period − comparison period), divided by the visits in the comparison period.

The data was analyzed using the STATA/MP (version 15, StataCorp LP) and statistical significance was set for two queues *p* < 0.05.

### Ethical aspects

The basic minimum set of data from the EDs of both centers, anonymized and collected retrospectively, was used. Due to the characteristics of this study, where patients do not participate and without access to medical history, there is no evaluation by a clinical research ethics committee, although it did have the approval of the management of the participating centers.

## Results

**Fig. 1:**
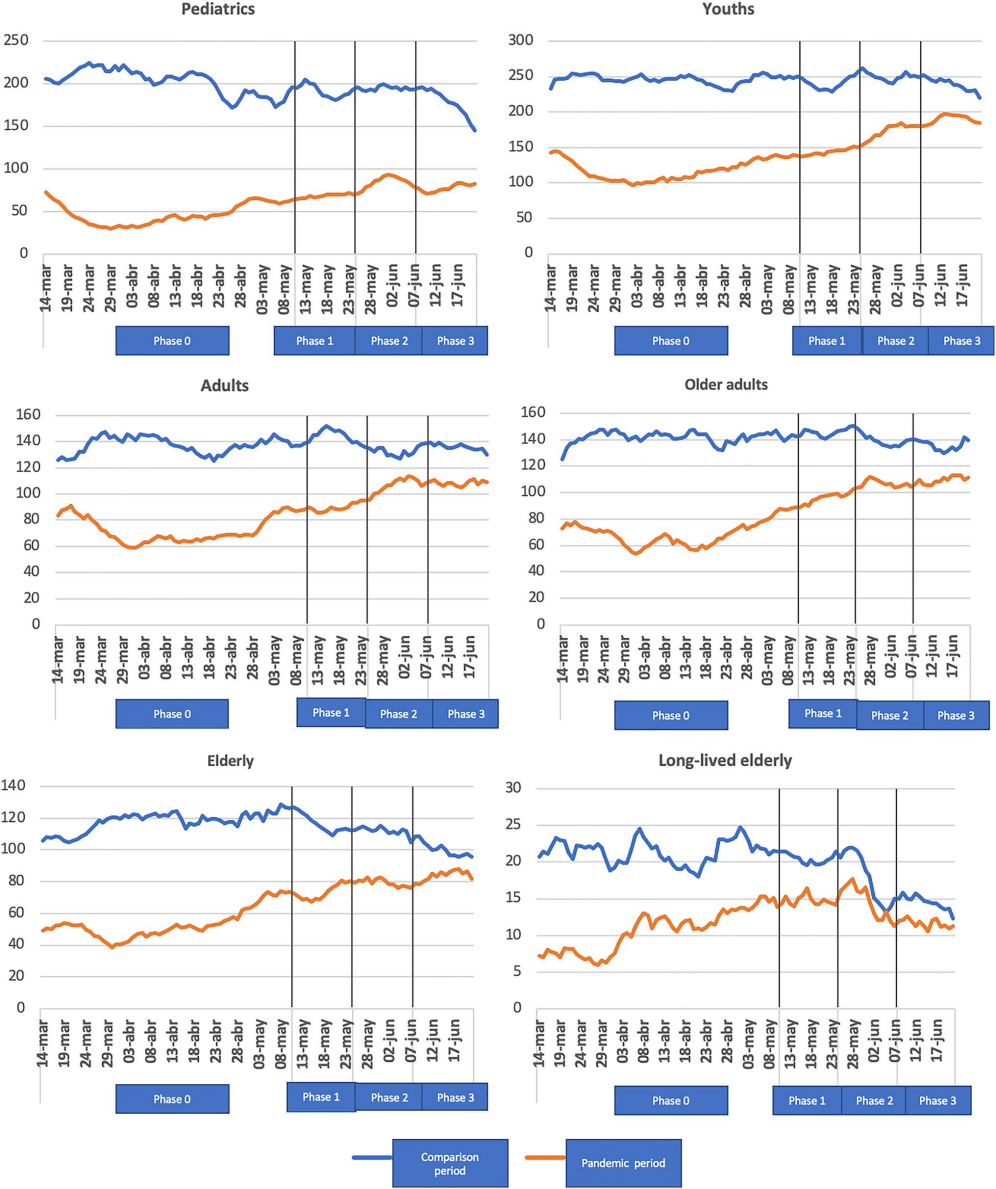
Evolution of visits to the EDs in the different phases of the SSA by age group compared to the previous year

Table 1 compares the characteristics of people who attended the EDs during the SSA and the comparison period. 44,373 visits were recorded in the pandemic period and 85,371 in the comparison period, which showed a significant decrease of 48% in the number of visits during the SSA. By specialties, a saturation of general emergencies was observed during the SSA, with a healthcare reduction of 44%, reaching 70% in pediatric emergencies. In the distribution by sex, in both periods analyzed, more women than men were attended the EDs. A greater reduction in male attendance was observed compared to female attendance during the pandemic period (49% vs 47%). The age of the patients was higher during the SSA, being 48.0 years (SD: 0.13), compared to 43.3 years (SD: 0.09) in the comparison period. An evident drop in care was observed in all age groups, the proportional difference was more evident in pediatric patients. A greater drop in attendances in the long-lived elderly in the HUGTiP compared to the HUMV stands out (56.5% vs 31.3%). The greatest decrease in the number of visits occurred in all age groups in phase 0 of the SSA (Fig. 1). In the later phases, the differences were not so clearly marked. By work shifts, there was an increase in attendance during the morning (45.0%) compared to a significant drop in the afternoon (38.1%) and night (16.7%) during the SSA (Table 1).

**Table 1:** Comparison of the characteristics of the people who attended the emergency department in two Spanish hospitals, from March 14 to June 21, 2019 (comparison period) and March 14 to June 21, 2020 (pandemic period or SSA)

	Both hospitals		*p*	HUMV		*p*	HUGTiP		*p*
	Pandemic period	Comparison period		Pandemic period	Comparison period		Pandemic period	Comparison period	
Total number of assists	44,373	85,371		25,149	45,020		19,224	40,351	
General EDs	34,429 (77,59)	61,080 (71,55)	< 0,001	19,270 (76,62)	31,717 (70,45)	< 0,001	15,159 (78,85)	29,363 (72,77)	< 0,001
Pediatric EDs	5338 (12,03)	17,499 (20,50)		3545 (14,10)	10,028 (22,27)		1793 (9,33)	7471 (18,52)	
Obstetrics and gynecology EDs	4606 (10,38)	6792 (7,96)		2334 (9,28)	3275 (7,27)		2272 (11,82)	3517 (8,72)	
Age [years(SD)]	48,03(0,13)	43,31(0,09)	< 0,001	47,99 (0,17)	43,45 (0,13)	< 0,001	48,08(0,19)	43,16 (0,13)	< 0,001
Age group [*n* (%)]									
Pediatric	5948 (13,40)	19,602 (22,96)	< 0,001	3764 (14,97)	10,554 (23,44)	< 0,001	2184 (11,36)	9048 (22,42)	< 0,001
Youth	13,962 (31,47)	24,466 (28,66)		7549 (30,02)	12,715 (28,24)		6413 (33,36)	11,751 (29,12)	
Adult	8495 (19,14)	13,747 (16,10)		4781 (19,01)	7145 (15,87)		3714 (19,32)	6602 (16,36)	
Older adult	8377 (18,88)	14,124 (16,54)		4561 (18,14)	7243 (16,09)		3816 (19,85)	6881 (17,05)	
Elderly	6398 (14,42)	11,451 (13,41)		3599 (14,31)	6061 (13,46)		2799 (14,56)	5390 (13,36)	
Long-lived elderly	1192 (2,69)	1981 (2,32)		895 (3,56)	1302 (2,89)		297 (1,55)	679 (1,68)	
Sex [*n* (%)]									
Male	20,013 (45,10)	39,301 (46,04)	0,001	11,301 (44,94)	20,712 (46,01)	0,006	8712 (45,32)	18,589 (46,07)	0,086
Female	24,360 (54,90)	46,070 (53,96)		13,848 (55,06)	24,308 (53,99)		10,512 (54,68)	21,762 (53,93)	
Weekday [*n* (%)]									
Monday–Friday	32,687 (73,66)	62,522 (73,24)	0,098	18,438 (73,32)	32,852 (72,97)	0,326	14,249 (74,12)	29,670 (73,53)	0,125
Saturday–Sunday	11,686 (26,34)	22,849 (26,76)		6711 (26,68)	12,168 (27,03)		4975 (25,88)	10,681 (26,47)	
Workshift [*n* (%)]									
08:00–15:00	11,641 (45,08)	20,910 (42,56)	< 0,001	2894 (43,78)	3642 (41,46)	0,014	8747 (45,52)	17,268 (42,79)	< 0,001
15:00–22:00	9855 (38,16)	19,773 (40,24)		2527 (38,23)	3472 (39,53)		7328 (38,14)	16,301 (40,40)	
22:00–08:00	4329 (16,76)	8452 (17,20)		1189 (17,99)	1670 (19,01)		3140 (16,34)	6782 (16,81)	

*EDs* emergency departments, *SD* standard deviation

**Fig. 2:**
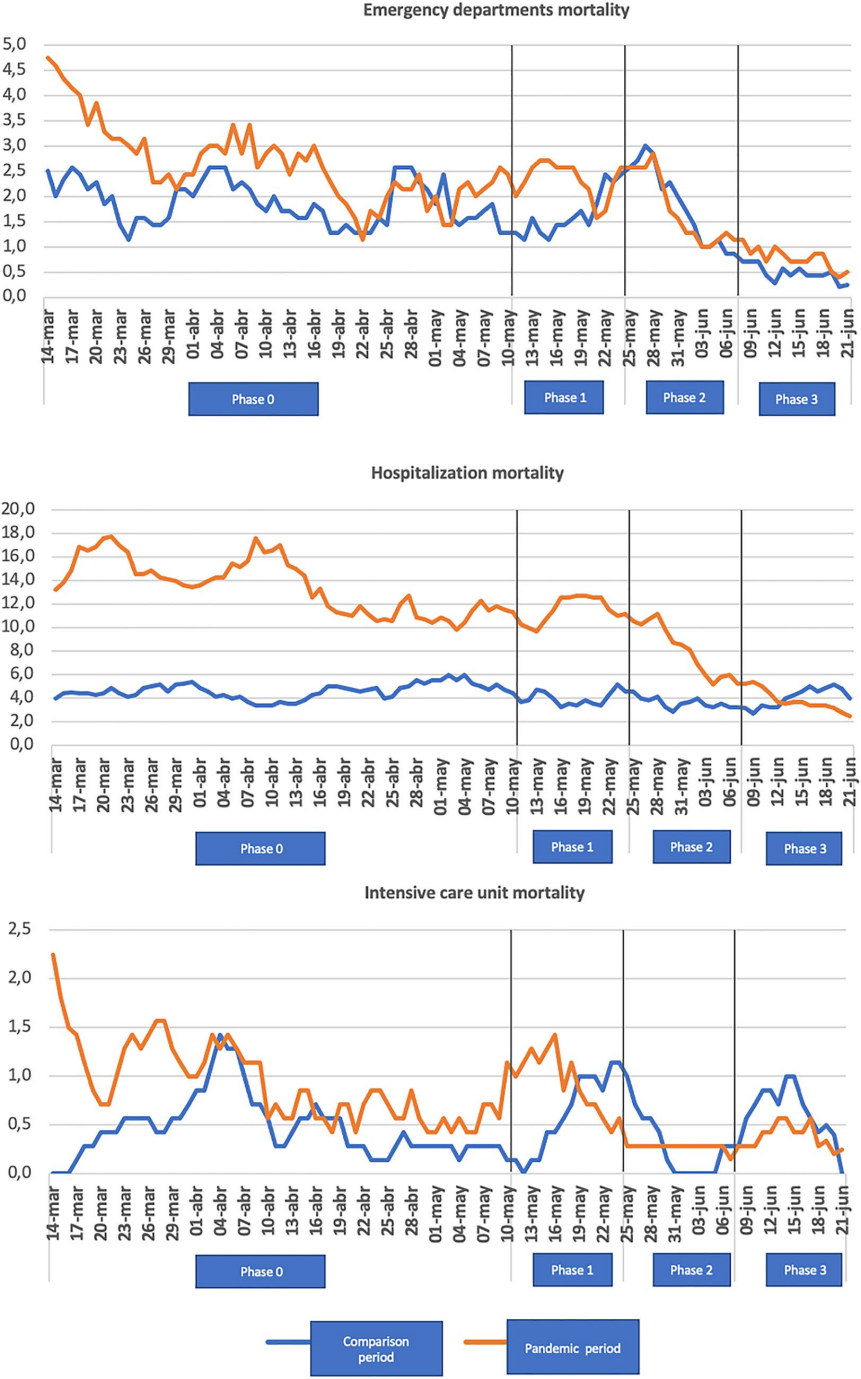
Weekly mortality in the EDs, hospitals, and intensive care unit in the different phases of the SSA compared to the previous year

Table 2 shows the average stay and the final destination of the patients. The mean stay in the EDs was not significant in the overall study; however, in the HUMV an increase in the average length of stay was observed during the SSA (6.3 h) compared to the previous year (4.1 h). When we studied the final destination of the patients, a drop in home discharges was observed in the study period, compared to an increase in hospital admissions. ICU admissions were higher during the pandemic period (4.8%) compared to the previous year (4.0%). There are differences between hospitals, highlighting a lower percentage of ICU admissions in the HUMV during the study period (3.5%) compared to the control period (5.1%). An increase in the percentage of patients who were discharged before being treated or who requested voluntary discharge was observed during the study period compared to the control year, without this difference being significant. The mean age of these patients was higher during the SSA, 44.3 years (SD 16.9), compared to 39.2 years (SD 19.9) during the comparison period (*p* < 0.05). Regarding deaths, an increase in patients who died in the EDs, hospital, and ICU was observed during the study period compared to the previous year. Figure 2 shows how this increase in mortality was higher in phases 0 and 1 of the SSA, estimating a mortality related to COVID-19 of 13%.

**Table 2:** Mean stays and final destination of patients attending the EDs during the EAE and the comparison period

	Both hospitals		*p*	HUMV		*p*	HUGTiP		*p*
	Pandemic period	Comparison period		Pandemic period	Comparison period		Pandemic period	Comparison period	
Total number of attendances	44,373	85,371		25,149	45,020		19,224	40,351	
Mean stay in the EDs—min (SD)	300,61 (60,01)	268,88 (44,37)	0,671	380,47 (6,94)	247,80 (7,58)	< 0,001	278,78 (72,98)	271,18 (50,36)	0,932
Discharge home [*n* (%)]	35,328 (79,62)	75,885 (88,89)	< 0,001	20,071 (79,81)	39,432 (87,59)	< 0,001	15,257 (79,36)	36,453 (90,34)	< 0,001
Hospital admission [*n* (%)]	9045 (20,38)	9486 (11,11)	< 0,001	5078 (20,19)	5588 (12,41)	< 0,001	3967 (20,64)	3898 (9,66)	< 0,001
ICU admission [*n* (%)]	355 (4,82)	385 (4,06)	< 0,001	145 (3,55)	287 (5,14)	< 0,001	210 (6,40)	98 (2,51)	< 0,001
Mean hospital stay [days(SD)]	8,87 (0,15)	8,06 (0,14)	< 0,001	8,01 (0,22)	8,14 (0,20)	0,683	9,75 (0,20)	7,96 (0,20)	< 0,001
EDs mortality [*n* (%)]	221 (0,57)	163 (0,19)	< 0,001	114 (0,58)	88 (0,20)	< 0,001	107 (0,56)	75 (0,19)	< 0,001
Hospital mortality [*n* (%)]	1110 (13,76)	431 (4,55)	< 0,001	800 (19,47)	239 (4,28)	< 0,001	310 (7,83)	192 (4,93)	< 0,001
ICU mortality [*n* (%)]	77 (0,17)	48 (0,06)	< 0,001	28 (0,11)	23 (0,05)	< 0,001	49 (0,25)	25 (0,06)	< 0,001
Discharge without being visited or voluntary discharge [*n* (%)]	281 (0,63)	477 (0,56)	0,095	236 (0,94)	362 (0,80)	0,063	45 (0,23)	115 (0,28)	0,262

*EDs* emergency departments, *SD* standard deviation, *ICU* intensive care unit

Table 3 shows the comparison by groups of pathologies during the SSA compared to the previous period, observing a drop in absolute numbers in all groups. When comparing the proportions, an increase was found in the following pathologies: infectious (A00–B99), neoplastic (C00–D49), endocrine–metabolic (E00–E89), vascular (I00–I99), genitourinary (N00–N99), gynecology—obstetric (O00–O00), due to external causes (V00–V99) and contact reasons (Z00–Z99), in the face of a drop in neurological pathology (G00–G99), ophthalmological (H00–H59), otorhinolaryngological (H69–H95), respiratory not related to COVID-19 (J00–J99), digestive (K00–K95), cutaneous (L00–L99), musculoskeletal (M00–M99), by signs and symptoms (R00–R99), and trauma and poisoning (S00–T88). In the gynecological–obstetric pathology study, an increase in the percentage of abortions during the study period compared to the comparison period stood out, from 0.41 to 0.73% (*p* < 0.001). It should be noted that during the SSA, a significant drop in respiratory pathology (from 3.83 to 0.65%) and trauma (4.41 to 1.46%) was observed in pediatric patients (*p* < 0.001).

**Table 3:** Diagnoses grouped by pathologies according to ICD-10 and time-dependent pathologies before and during the state of alarm

ICD-10 diagnosis	Both hospitals		% difference*	*p*	HUMV		% difference *	*p*	HUGTiP		% difference*	*p*
	Pandemic period	Comparison period			Pandemic period	Comparison period			Pandemic period	Comparison period		
A00–B99	1137 (2,56)	2025 (2,37)	− 43,85%	0,035	537 (2,14)	1032 (2,29)	− 47,96%	0,177	600 (3,12)	993 (2,46)	− 39,57%	< 0,001
C00–D49	618 (1,39)	688 (0,81)	− 10,17%	< 0,001	322 (1,28)	383 (0,85)	− 15,92%	< 0,001	296 (1,54)	305 (0,76)	− 2,95%	< 0,001
D50–D89	220 (0,50)	359 (0,42)	− 38,71%	0,054	107 (0,43)	204 (0,45)	− 47,54%	0,597	113 (0,59)	155 (0,38)	− 27,09%	0,001
E00–E89	395 (0,89)	509 (0,60)	− 22,39%	< 0,001	231 (0,92)	284 (0,63)	− 18,66%	< 0,001	164 (0,85)	225 (0,56)	− 27,11%	< 0,001
F01–F99	1261 (2,84)	2491 (2,92)	− 49,37%	0,438	717 (2,85)	1179 (2,62)	− 39,18%	0,069	544 (2,83)	1312 (3,25)	− 58,53%	0,006
G00–G99	797 (1,80)	2291 (2,68)	− 65,21%	< 0,001	407 (1,62)	1205 (2,68)	− 66,22%	< 0,001	390 (2,03)	1086 (2,69)	− 64,08%	< 0,001
H00–H59	2420 (5,45)	6269 (7,34)	− 61,39%	< 0,001	882 (3,51)	2056 (4,57)	− 57,10%	< 0,001	1538 (8,00)	4213 (10,44)	− 63,49%	< 0,001
H60–H95	318 (0,72)	1331 (1,56)	− 76,10%	< 0,001	252 (1,00)	849 (1,89)	− 70,31%	< 0,001	66 (0,34)	482 (1,19)	− 86,30%	< 0,001
I00–I99	2504 (5,64)	3765 (4,41)	− 33,49%	< 0,001	1368 (5,44)	2081 (4,62)	− 34,26%	< 0,001	1136 (5,91)	1684 (4,17)	− 32,54%	< 0,001
J00–J99	3545 (7,99)	7444 (8,72)	− 52,37%	< 0,001	2487 (9,89)	4398 (9,77)	− 43,45%	0,608	1058 (5,50)	3046 (7,55)	− 65,26%	< 0,001
K00–K95	2574 (5,80)	5226 (6,12)	− 50,74%	0,021	1619 (6,44)	3254 (7,23)	− 50,24%	< 0,001	955 (4,97)	1972 (4,89)	− 51,57%	0,670
L00–L99	959 (2,16)	2143 (2,51)	− 55,24%	< 0,001	535 (2,13)	1085 (2,41)	− 50,69%	0,017	424 (2,21)	1058 (2,62)	− 59,92%	0,002
M00–M99	2804 (6,32)	8150 (9,55)	− 65,59%	< 0,001	1570 (6,24)	4377 (9,72)	− 64,13%	< 0,001	1234 (6,42)	3773 (9,35)	− 67,29%	< 0,001
N00–N99	2732 (6,16)	5092 (5,96)	− 46,34%	0,167	1511 (6,01)	2603 (5,78)	− 41,95%	0,221	1221 (6,35)	2489 (6,17)	− 50,94%	0,387
O00–O99	2294 (5,17)	2783 (3,26)	− 17,57%	< 0,001	1387 (5,52)	1635 (3,63)	− 15,16%	< 0,001	907 (4,72)	1148 (2,85)	− 20,99%	< 0,001
P00–P96	94 (0,21)	175 (0,20)	− 46,28%	0,797	70 (0,28)	84 (0,19)	− 16,66%	0,013	24 (0,12)	91 (0,23)	− 73,62%	0,009
Q00–Q99	16 (0,04)	39 (0,05)	− 58,97%	0,424	10 (0,04)	13 (0,03)	− 23,07%	0,445	6 (0,03)	26 (0,06)	− 76,92%	0,102
R00–R99	7028 (15,84)	14,949 (17,51)	− 52,98%	< 0,001	3653 (14,53)	6232 (13,84)	− 41,38%	0,013	3375 (17,56)	8717 (21,60)	− 61,28%	< 0,001
S00–T88	6303 (14,20)	14,298 (16,75)	− 55,91%	< 0,001	4372 (17,38)	8990 (19,97)	− 51,36%	< 0,001	1931 (10,04)	5308 (13,15)	− 63,62%	< 0,001
V00–Y99	163 (0,37)	219 (0,26)	− 25,57%	< 0,001	161 (0,64)	208 (0,46)	− 22,59%	0,002	2 (0,01)	11 (0,03)	− 81,81%	0,193
Z00–Z99	2077 (4,68)	2599 (3,04)	− 20,08%	< 0,001	1463 (5,82)	1380 (3,07)	6,01%	< 0,001	614 (3,19)	1219 (3,02)	− 49,63%	0,253
U00–U48	1808 (4,07)				481 (1,91)				1327 (6,90)			
Time-dependent pathologies												
Cardiovascular diseases	654 (1,47)	819 (0,96)	− 20,14%	< 0,001	310 (1,23)	358 (0,80)	− 13,40%	< 0,001	344 (1,79)	461 (1,14)	− 25,37%	< 0,001
Myocardial infarction	168 (0,38)	218 (0,26)	− 22,93%	< 0,001	105 (0,42)	121 (0,27)	− 13,22%	< 0,001	63 (0,33)	97 (0,24)	− 35,05%	0,054
Stroke	486 (1,10)	601 (0,70)	− 19,13%	< 0,001	205 (0,82)	237 (0,53)	− 13,50%	< 0,001	281 (1,46)	364 (0,90)	− 22,80%	< 0,001
Sepsis	136 (0,31)	202 (0,24)	− 32,67%	0,019	86 (0,34)	133 (0,30)	− 35,33%	0,289	50 (0,26)	69 (0,17)	− 27,53%	0,023
Intoxications	256 (0,58)	507 (0,59)	− 49,50%	0,705	136 (0,54)	304 (0,68)	− 55,26%	0,03	120 (0,62)	203 (0,50)	− 40,88%	0,06

*ICD-10* International Statistical Classification of Diseases and Related Health Problems 10th Revision. *A00–B99* certain infectious and parasitic diseases. *C00–D49* neoplasms. *D50–D89* diseases of the blood and blood-forming organs and certain disorders involving the immune mechanism. *E00–E89* endocrine, nutritional, and metabolic diseases. *F00–F99* mental and behavioral disorders. *G00–G99* diseases of the nervous system. *H00–H59* diseases of the eye and adnexa. *H69–H95* diseases of the ear and mastoid process. *I00–I99* diseases of the circulatory system. *J00–J99* diseases of the respiratory system. *K00–K95* diseases of the digestive system. *L00–L99* diseases of the skin and subcutaneous tissue. *M00–M99* diseases of the musculoskeletal system and connective tissue. *N00–N99* diseases of the genitourinary system. *O00–O00* pregnancy, childbirth and the puerperium. *P00–P96* certain conditions originating in the perinatal period. *Q00–Q99* congenital malformations, deformations and chromosomal abnormalities. *R00–R99* symptoms, signs, and abnormal clinical and laboratory findings, not elsewhere classified. *S00–T88* injury, poisoning, and certain other consequences of external causes. *V00–V98* external causes of morbidity and mortality. *Z00–Z99* factors influencing health status and contact with health services. *U00–U85* codes for special purposes

**Fig. 3:**
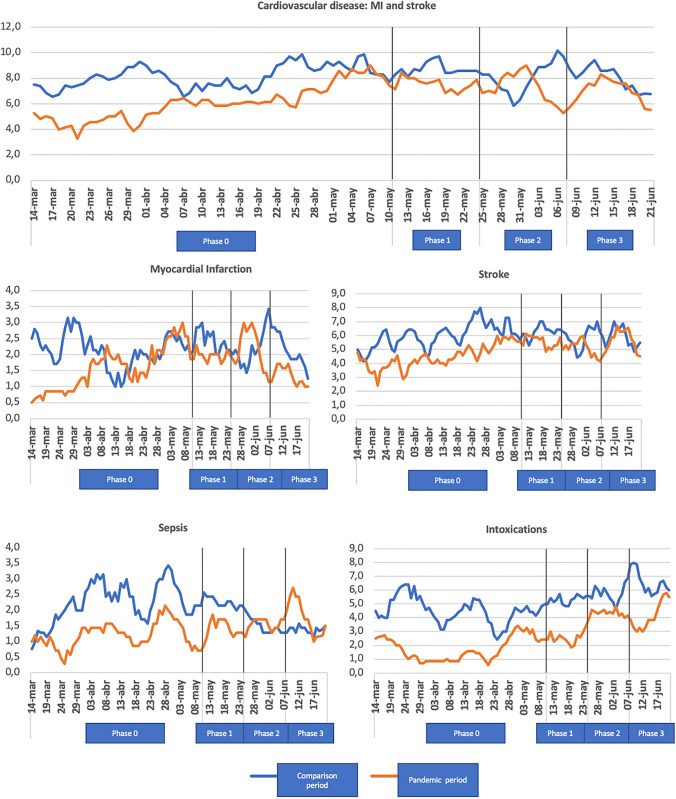
Weekly time-dependent pathologies in the different phases of SSA compared to the previous year

Figure 3 shows the time-dependent pathologies. A decrease in the absolute number of cardiovascular diseases was observed, both MI and stroke, and sepsis and intoxications, especially in phase 0 of the SSA. However, Table 3 shows a relative increase in both MI and stroke when the percentages are compared by period. There are no differences in mortality secondary to cardiovascular diseases. Sepsis showed a percentage increase during the pandemic period from 0.24 to 0.31% in both hospitals, with this increase differing in the individual analysis. No differences were found in the overall study in patients who presented intoxicated, but in the individual study, the HUMV showed a significant drop during in the SSA from 0.68 to 0.54% (Table 3).

## Discussion

This study has shown a decrease in visits to two Spanish tertiary EDs during the SSA. Multiple studies worldwide have shown a drop in attendance at EDs during the first stage of the COVID-19 pandemic [2, 12–21]. This drop has occurred in all age groups, being more marked in pediatric and young patients, associated with a decrease in visits for respiratory and traumatic pathologies. Other studies have also observed this decrease [12–22]. The reasons for the decrease in the number of patients seen during this first period of the pandemic are multifactorial and are derived from restrictions on outdoor activities [21, 22]; a lower number of viral respiratory infections not related to COVID-19 due to the use of a mask and hand hygiene [23, 24]; and importantly the probable fear of the population of becoming infected by SARS-CoV-2 [6, 25]. When we evaluated the different phases of the SSA, the greatest drop in attendance in the EDs occurred in phase 0, the same result that Montero-Pérez reflected in his study [13]. The fact that the different phases of the SSA have different attendance patterns in the EDs seems to be linked to the progressive lifting of the different restrictions. In our study, less attendance was observed in the afternoon and night shifts, compared to an increase in attendance during the morning, probably related to the time constraints and mobility limitations, a trend already observed in Italy by Veronica Ojetti [20].

In this study, we observed an increase in care overload in medical emergencies. Souza showed a lower number of attendances in the different specialties; however, when comparing the proportions, he observed a significant increase in the volume of patients attended by internal medicine [18]. This may be due to the change in the patterns of patients who attended the SSA, with those with COVID-19 predominating over those with other diseases**.** When analyzing the final diagnoses of the patients who attended during the SSA, less severe pathologies were less prevalent, such as those related to signs and symptoms, musculoskeletal, ophthalmological, and/or otorhinolaryngological, results previously observed during SARS or MERS epidemics [4, 5]. Likewise, Hartnett reported an increase in visits due to exposure to infectious diseases, COVID-19, other signs, and pneumonias, compared to a decrease in the number of visits due to pain from abdominal and digestive symptoms, musculoskeletal pain, high blood pressure, and nausea or vomiting during the initial phase of the COVID-19 pandemic in the USA [12].

In our study, pediatric and gynecological–obstetric emergencies were clearly reduced, associated with an increase in cases of abortions. Similar results were found by Spurlin in New York, derived from less access to routine prenatal care during the SSA or from the fear generated by COVID-19 admissions to the EDs, which could have led to the development of undiagnosed pregnancy complications [26]. Moreover, we observed a significant drop in traumatic pathology at all ages, a trend that was previously evidenced by Reschen [19], Comelli [21] and Núñez [27], probably secondary to restrictions in place that led to fewer outdoor activities, less sports practices, and a lower number of traffic accidents [10].

When we assess the patients with life-threatening pathologies who attended the EDs, we observed a decrease in the absolute number of patients diagnosed with cardiovascular disease, both MI and stroke. In Spain, the Interventional Cardiology Association of the Spanish Society of Cardiology showed in a multicenter study a decrease in the number of patients with ST-segment elevation AMI, as well as an increase in hospital mortality [25]. In the USA, Lange found a greater decrease in EDs visits for MI and stroke, with percentage differences of −23% and −20%, respectively [28]. In France, Mesnier found a decrease in admissions for MI [6]. Wong showed higher rates of out-of-hospital cardiac arrest, probably in relation to the delay in the care of patients with MI [8]. In Norway, Kristoffersen observed a lower number of admissions due to stroke, as well as patients with greater severity according to the NIHSS scale [9]. However, in the UK, Reschen found no changes in patients presenting to EDs with stroke [19]. This decrease in visits to the EDs due to life-threatening pathologies could be related to multiple factors secondary to the pandemic, either due to fear of exposure to SARS-CoV-2 infection or the effect of audiovisual media advising not to overload the EDs or public. Therefore, it is very important to reinforce the health education of the population, so that they can detect, as a priority, alarm data of serious cardiovascular pathologies [6–9, 12, 25, 28].

In our study, no differences were found between patients who left the EDs without being seen or who requested voluntary discharge during the study period. These results reflect good use of the health system, especially when greater collaboration from society is expected due to the SSA. In the non-pandemic period, Mataloni described an abandonment of the ED by patients before being visited by the doctor or during treatment in more than 13% [29].

In the mortality study, we evidenced a global increase in the visits to EDs, hospitals, and ICU. Note that 87% of all deaths were not due to COVID-19. All of this is consistent with an increase in the Spanish mortality rate during the SSA according to updated data from the National Institute of Statistics [30]. In Korea, Kang showed an increase in ED mortality in patients older than 60 years during the COVID-19 pandemic, but not in pediatric patients or patients younger than 60 years [14]. In Thailand, Wongtanasarasin demonstrated an increase in overall mortality during the local lockdown period [17]. This reflects a greater severity of the patients who attended the EDs during the SSA, as well as a probable delay in diagnoses, secondary to the restrictions and the population’s fear of a possible SARS-CoV-2 infection.

### Strengths and limitations

The main limitation of this study is its cross-sectional nature, which does not allow for checking the causality criterion of the results obtained. However, as it was carried out in two hospitals with different incidences of COVID-19 which displayed similar results, it shows that the results of the study represent the impact the COVID-19 pandemic has on the EDs. On the other hand, it may be that the real number of patients with COVID-19 is greater than that described, both because the polymerase chain reaction for SARS-CoV-2 is not completely sensitive and because some patients may not have been tested if they had attended for another reason.

## Conclusion

In conclusion, our study has shown a decrease in global attendance at EDs during the initial phase of the COVID-19 pandemic. In addition, there has been evidence of a decrease in care for serious pathologies such as cardiovascular diseases. It is therefore necessary to educate the population to consult before alarm data of pathologies with high vital risk, given the high morbidity and mortality rate if the diagnosis is delayed.
